# Clinical associations of right ventricular strain in community-acquired pneumonia: a prospective cohort study

**DOI:** 10.1186/s44348-026-00074-9

**Published:** 2026-06-01

**Authors:** Eren Ozan Bakir, Mutlu Onur Gucsav, Ahmet Anil Baskurt

**Affiliations:** 1 Department of Cardiology, Cigli Training and Research Hospital, IzmirBakircayUniversity, Izmir, Turkey; 2 Department of Chest Diseases, Cigli Training and Research Hospital, IzmirBakircayUniversity, Izmir, Turkey

**Keywords:** Community-acquired pneumonia, Echocardiography, Global longitudinal strain, Hospitalization, Prognosis

## Abstract

**Background:**

Pneumonia remains a major cause of morbidity and mortality worldwide, and right ventricular dysfunction may contribute to adverse clinical outcomes. Right ventricular global longitudinal strain (RVGLS) is an emerging echocardiographic marker for assessing right ventricular function, but its prognostic and clinical utility in pneumonia has not been fully clarified.

**Methods:**

Eighty patients hospitalized with pneumonia underwent echocardiographic assessment, including RVGLS measurement via 2D speckle tracking echocardiography. Patients were categorized into two groups: reduced RVGLS (absolute value < 20%) and normal RVGLS (absolute value ≥ 20%). Demographic, clinical, laboratory, and echocardiographic data were compared, and associations with in-hospital outcomes were analyzed.

**Results:**

Twenty-eight patients (35%) had reduced RVGLS. Compared with the normal RVGLS group, patients with reduced RVGLS had significantly higher N-terminal pro–brain natriuretic peptide and troponin levels. Echocardiographically, patients with reduced RVGLS demonstrated lower tricuspid annular plane systolic excursion and right ventricular ejection fraction, higher pulmonary artery pressures, and larger right ventricular and pulmonary artery diameters, although most diameter measurements remained within reference value thresholds (all *P* < 0.05). Prolonged hospitalization > 7 days (50.0% vs. 28.8%, *P* = 0.044) and > 10 days (21.4% vs. 5.8%, *P* = 0.029), as well as longer duration of oxygen therapy (4.30 days vs. 2.60 days, *P* = 0.043), were significantly more common in the reduced RVGLS group. Intensive care unit admission and short-term mortality did not differ significantly between groups.

**Conclusions:**

Reduced RVGLS in pneumonia patients is associated with markers of disease severity, longer hospital stays, and extended oxygen therapy requirements, even without overt right ventricular failure. RVGLS assessment may assist in identifying patients at risk for a complicated clinical course.

**Graphical Abstract:**

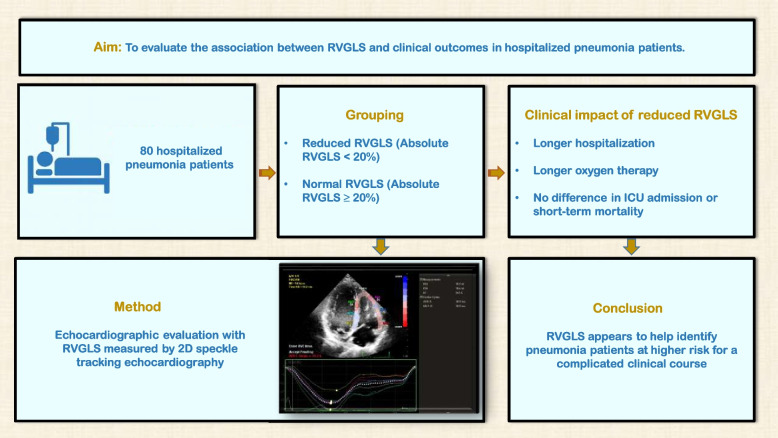

## Background

Community-acquired pneumonia (CAP) is the most common cause of infection-related deaths globally among individuals over 60 years of age. According to the World Health Organization, lower respiratory tract infections result in 3.2 million fatalities annually [1]. Predicting prognosis of this highly morbid disease is critical, as it not only helps prevent disease progression through early interventions but may also reduce morbidity, mortality, and prolonged hospitalizations in affected patients.

Previous studies suggested predictors associated with short-term and long-term mortality in patients hospitalized for CAP. Key factors include advanced age, a high Charlson Comorbidity Index score, elevated respiratory rate, and hypothermia [2]. While CAP mortality rates are higher in patients with cardiac disease, cardiac effects secondary to CAP may also contribute to prognosis. The cardiac impact observed in patients without preexisting cardiac disease during follow-up may indicate disease severity and thereby provide valuable prognostic information for clinicians. Echocardiographic strain analysis, a sensitive modality for evaluating both systolic and diastolic ventricular function, has emerged as a valuable tool for predicting disease progression in patients with CAP [3].

Studies conducted during the COVID-19 pandemic demonstrated that biventricular systolic and diastolic dysfunctions in patients hospitalized with COVID-19 pneumonia are associated with prognosis. In particular, strong associations were found in patients who exhibited markedly reduced right ventricular global longitudinal strain (RVGLS) values and adverse outcomes, such as the need for intubation and mortality, compared to those with normal values [4]. Although the relationship between changes in right ventricular strain values and prognosis has been established in patients with COVID-19 pneumonia, there is limited evidence on this relationship in individuals with CAP. In this study, we investigated the clinical course and outcomes of patients with reduced versus normal RVGLS.

## Methods

### Ethics statement

This study was approved by the Izmir Bakircay University Ethics Committee (No. 1666; Research No. 1646). The study was conducted in accordance with the Declaration of Helsinki and its subsequent amendments. Written informed consents were obtained from the patient(s).

### Study design

This prospective cohort study was conducted at the Bakircay University Cigli Training and Research Hospital between July 15, 2024, and July 15, 2025. The study included 97 adult patients who were hospitalized in the pulmonology ward with diagnoses of CAP. Eligible participants underwent transthoracic echocardiographic evaluation within the first 24 h of hospital admission. Patients were excluded if they had respiratory diseases affecting the right ventricular (RV) strain (acute or chronic pulmonary embolism [*n* = 1], pulmonary hypertension [*n* = 0], chronic obstructive pulmonary diseases [*n* = 2], diffuse parenchymal lung diseases [*n* = 0]), cardiovascular diseases (obstructive coronary artery diseases [*n* = 2], prior myocardial infarction [*n* = 1], atrial fibrillation [*n* = 4], cardiomyopathy [*n* = 0], valvular stenosis [*n* = 2], severe valvular regurgitation [*n* = 2], heart failure with reduced or preserved ejection fraction [*n* = 2], or diastolic dysfunction as defined by the diastolic dysfunction algorithm [*n* = 1]) [5].

Based on RVGLS values, patients were categorized into two groups: reduced RVGLS group, defined as an absolute RVGLS of < 20%, and normal RVGLS group, defined as an absolute RVGLS of ≥ 20%. Patients were followed prospectively for a period of 30 days. Comparative analyses were performed between the two groups based on predefined clinical and laboratory parameters.

### Definition of RV strain

RV strain analysis is an advanced echocardiographic method that assesses myocardial function beyond conventional echocardiographic parameters. It quantifies the percentage change in myocardial fiber length during the cardiac cycle, with more negative values indicating better longitudinal shortening. Two-dimensional speckle tracking echocardiography is the primary technique used, as it tracks naturally occurring acoustic markers within the myocardium to evaluate motion independent of Doppler angle. An apical four-chamber view optimized for the RV is obtained For RV strain assessment, ensuring clear visualization of the endocardial borders. The endocardial border is manually traced, and software generates a segmented region of interest. RVGLS uses average values of peak systolic strain from both free wall and septal segments, while RV free wall strain only uses free wall segments. Unlike conventional echocardiographic methods, strain imaging allows early detection of subtle myocardial dysfunction. Lower (less negative) strain values indicate impaired ventricular function and are associated with adverse outcomes in conditions such as pulmonary hypertension, acute respiratory distress syndrome, and pneumonia [6, 7].

### Echocardiographic measurements

All echocardiographic measurements were performed within the first 24 h of hospitalization using a Philips EPIQ 7G echocardiography system (Philips) by a single physician to minimize interobserver variability and eliminate device-related inconsistencies. Measurements were obtained in accordance with current echocardiography guidelines [6]. RV basal and mid-cavity diameters were measured from the apical four-chamber view, while the parasternal long-axis view was used for standard RV dimension measurement. Pulmonary artery systolic pressure was calculated by applying the Bernoulli equation to the tricuspid regurgitation velocity, with an additional estimated right atrial pressure of 5 cmH₂O. No patient required assignment of right atrial pressure of 10 or 15 cmH₂O, as individuals with clinical signs of volume overload or elevated brain natriuretic peptide (BNP) levels suggestive of heart failure with preserved ejection fraction had already been excluded. RV strain analysis was performed using 2D AutoStrain software (QLab, Philips Healthcare). Adequate image acquisition was ensured to clearly delineate the lateral free wall and interventricular septum of the RV, and RVGLS was calculated using a six-segment region of interest. To define the cutoff value for reduced RV function, we predefined reduced RVGLS as an absolute global RV longitudinal strain < 20% (i.e., RVGLS > –20%). This threshold was chosen based on previous studies in patients with pneumonia and in large cohorts of healthy volunteers, in which RVGLS values around –20% approximated the lower limit of normal and were associated with adverse outcomes [4].

### Statistical analysis

Statistical analyses were performed using IBM SPSS ver. 26.0 (IBM Corp). Descriptive statistics for continuous variables were presented as mean ± standard deviation or median and interquartile range (IQR), while categorical variables were expressed as frequencies and percentages. The normality of data distribution was assessed by the Kolmogorov–Smirnov test. For group comparisons, the independent samples t-test was used for normally distributed continuous variables, and the Mann–Whitney U-test was applied for non-normally distributed variables. Categorical variables were compared using the chi-square test or the Fisher exact test, as appropriate. A *P*-value of < 0.05 was considered statistically significant.

The sample size was calculated a priori using GPower ver. 3.1.9.7 (Heinrich Heine University Düsseldorf). We used the t-test family (“difference between two independent means”) with a two-sided α of 0.05, a statistical power of 95%, and an allocation ratio of 1:1. The expected effect size was derived from Krishnamoorthy et al. [3], based on the relationship between RVGLS and clinical outcomes in patients with COVID-19 pneumonia. Based on the reported difference in RVGLS values between outcome groups in that study, the calculated effect size (Cohen d) was 2.00. Using this effect size, the minimum total sample size required for adequate power was 14 patients. A sensitivity analysis using a conventional large effect size (d = 0.80) showed that approximately 70 participants (35 per group) would be sufficient. Ultimately, 80 eligible patients were enrolled. Due to the natural clinical distribution of the studied phenotypes, groups were not equally sized and comprised 28 and 52 participants, respectively.

## Results

A total of 80 patients were included in the study, of whom 28 (35%) presented with reduced RVGLS and 52 (65%) with normal RVGLS. Baseline demographic characteristics, including age and sex distribution, are summarized in Table 1. Smoking status differed significantly between groups, although the overall prevalence of smoking was similar (39.3% vs. 38.5%). No significant differences in vital signs were observed between groups. Compared to patients with normal RVGLS, those with reduced RVGLS had significantly lower hemoglobin levels (11.69 ± 2.12 g/dL vs. 12.86 ± 2.12 g/dL, *P* = 0.022) and higher N-terminal pro-BNP (NT-proBNP; 1,167.5 pg/mL [IQR, 532.5–2,371.3 pg/mL] vs. 369.0 pg/mL [IQR, 101.0–1,039.0 pg/mL], *P* = 0.014) and troponin levels (26.0 ng/L [IQR, 14.0–43.0 ng/L] vs. 11.0 ng/L [IQR, 6.8–16.0 ng/L], *P* < 0.001). No significant differences in other laboratory parameters were observed between groups (Table 2).

**Table 1:** Baseline characteristics and medical history

Characteristic	Total (*n* = 80)	Reduced RVGLS (*n* = 28)	Normal RVGLS (*n* = 52)	*P*-value
Age (yr)	65.55 ± 16.79	72.89 ± 12.19	61.6 ± 17.67	0.003^a^
Sex				0.367
Female	29 (36.2)	12 (42.9)	17 (32.7)	
Male	51 (63.8)	16 (57.1)	35 (67.3)	
Smoking status				0.011
Nonsmoker	31 (38.8)	11 (39.3)	20 (38.5)	
Ex-smoker	26 (32.5)	14 (50.0)	12 (23.0)	
Current smoker	23 (28.8)	3 (10.7)	20 (38.5)	
Comorbidity				
Hypertension	33 (41.2)	13 (46.4)	20 (38.5)	0.278
Diabetes mellitus	17 (21.2)	8 (28.6)	9 (17.3)	0.240
Chronic kidney disease	5 (6.2)	4 (14.3)	1 (1.9)	0.029
Vital sign				
SBP (mmHg)	133.41 ± 24.93	134.46 ± 31.11	132.85 ± 21.22	0.784^a^
DBP (mmHg)	72.86 ± 11.81	73.93 ± 12.55	72.29 ± 11.48	0.557^a^
Heart rate (bpm)	91.52 ± 14.57	90.18 ± 15.61	92.25 ± 14.08	0.548^a^
O_2_ saturation (%)	92.69 ± 4.17	92.75 ± 4.40	92.65 ± 4.06	0.922^a^

Values are presented as mean ± standard deviation or number (%)

*bpm* beats per minute, *DBP *diastolic blood pressure, *RVGLS* right ventricular global longitudinal strain, *SBP* systolic blood pressure

^a^Independent samples t-test

**Table 2:** Comparisons of blood parameters between RVGLS groups (*n* = 80)

Parameter	Reduced RVGLS (*n* = 28)	Normal RVGLS (*n* = 52)	*P*-value
Hemoglobin (g/dL)	11.69 ± 2.12	12.86 ± 2.12	0.022
Hematocrit (%)	35.84 ± 5.79	38.76 ± 7.27	0.070
WBC count (10^3^/µL)	13.64 ± 7.59	12.93 ± 5.42	0.630
Neutrophil count (10^3^/µL)	11.39 ± 6.99	10.64 ± 5.42	0.593
Lymphocyte count (10^3^/µL)	1.31 ± 0.66	1.30 ± 0.73	0.622
C-reactive protein (mg/L)	163.39 ± 112.78	167.94 ± 117.72	0.868
Serum creatinine (mg/dL)	1.21 ± 0.70	1.06 ± 0.64	0.353
NT-proBNP (pg/mL)	1,167.5 (532.5–2,371.3)	369.0 (101.0–1,039.0)	0.014^a^
Cardiac troponin (ng/L)	26.0 (14.0–43.0)	11.0 (6.8–16.0)	< 0.001^a^

Values are presented as mean ± standard deviation or median (interquartile range)

*NT-proBNP* N-terminal pro–brain natriuretic peptide, *RVGLS* right ventricular global longitudinal strain, *WBC* white blood cell count

^a^Mann-Whitney U-test

Echocardiographic characteristics of the two groups are summarized in Table 3. Significant differences in RV function were noted in the reduced RVGLS group, although most values remained within normal limits. These included lower tricuspid annular plane systolic excursion (TAPSE) values (20 mm [IQR, 18–22 mm] vs. 22 mm [IQR, 19–25 mm], *P* = 0.002), lower RV ejection fraction (RVEF; 44.0% [IQR, 41.0%–48.0%] vs. 49.5% [IQR, 45.0%–53.0%], *P* < 0.001), and higher resting heart rate (96.5 beats per minute [bpm; IQR, 80.0–106.3 bpm] vs. 81.5 bpm [IQR, 71.3–94.0 bpm], *P* = 0.007). The RV diameter measured in the parasternal long-axis view was also greater in the reduced RVGLS group (28.0 mm [IQR, 25.3–29.0 mm] vs. 25.0 mm [IQR, 23.0–27.0 mm], *P* = 0.005), as was the pulmonary artery diameter (23.92 ± 2.56 mm vs. 22.72 ± 2.22 mm, *P* = 0.040) (Table 3).

**Table 3:** Comparisons of echocardiographic parameters between RVGLS groups (*n* = 80)

Parameter	Reduced RVGLS (*n* = 28)	Normal RVGLS (*n* = 52)	*P*-value
Aortic diameter (mm)	33.79 ± 2.74	32.35 ± 3.75	0.077
Ascending aorta diameter (mm)	37.0 (34.5–39.0)	34.0 (31.0–35.0)	0.004
Left atrial diameter (mm)	37.18 ± 4.30	36.13 ± 3.91	0.275
LVEDD (mm)	46.39 ± 4.00	44.83 ± 4.95	0.154
LVESD (mm)	24.89 ± 5.12	23.41 ± 4.33	0.183
Posterior wall thickness (mm)	8.79 ± 1.03	8.55 ± 1.01	0.325
Interventricular septum thickness (mm)	10.46 ± 1.37	9.65 ± 1.34	0.012
RV diameter (mm)			
Parasternal long axis	28.0 (25.3–29.0)	25.0 (23.0–27.0)	0.005^a^
Basal segment	36.29 ± 3.98	36.06 ± 4.22	0.815
Mid-cavity	28.93 ± 3.51	28.67 ± 4.01	0.777
Pulmonary artery diameter (mm)	23.92 ± 2.56	22.72 ± 2.22	0.040
Aortic valve maximum velocity (m/sec)	1.54 ± 0.32	1.48 ± 0.22	0.307
Pulmonary valve maximum velocity (m/sec)	1.04 ± 0.18	0.94 ± 0.14	0.008
Mitral E (cm/sec)	53.0 (47.5–72.8)	66.0 (57.0–81.0)	0.013^a^
Mitral A (cm/sec)	79.0 (69.8–84.0)	84.0 (70.0–92.0)	0.229^a^
Lateral e’ (cm/sec)	12.07 ± 8.15	10.88 ± 2.31	0.335
Septal e’ (cm/sec)	10.33 ± 14.79	7.88 ± 1.52	0.242
Aortic regurgitation (n)	0.86 ± 0.36	0.86 ± 0.36	> 0.999
Mitral regurgitation (n)	1.00 ± 0.45	0.97 ± 0.33	0.753
TR (n)	1.25 ± 0.52	1.10 ± 0.36	0.131
LVEF (%)	61.61 ± 5.00	62.19 ± 4.03	0.571
sPAP (mmHg)	30.0 (24.3–35.5)	25.0 (19.0–29.8)	0.015^a^
TR maximum velocity (m/sec)	2.5 (2.1–2.9)	2.2 (1.9–2.4)	0.011^a^
TAPSE (mm)	20 (18–22)	22 (19–25)	0.002^a^
RVEF (%)	44.0 (41.0–48.0)	49.5 (45.0–53.0)	< 0.001^a^
RVGLS (%)	18.2 (16.9–18.9)	23.6 (21.9–26.3)	< 0.001^a^
Heart rate^b^ (bpm)	96.5 (80.0–106.3)	81.5 (71.3–94.0)	0.007^a^

Values are presented as mean ± standard deviation or median (interquartile range)

*bpm* beats per minute, *LVEDD* left ventricular end-diastolic diameter, *LVEF* left ventricular ejection fraction, *LVESD* left ventricular end-systolic diameter, *RV* right ventricular, *RVEF* right ventricular ejection fraction, *RVGLS* right ventricular global longitudinal strain, *sPAP* systolic pulmonary artery pressure, *TAPSE* tricuspid annular plane systolic excursion, *TR* tricuspid regurgitation

^a^Mann-Whitney U-test. ^b ^Recorded at the time of RV strain measurement

The mean length of hospitalization was 6.25 days. The mean length of hospitalization was longer in the reduced RVGLS group compared to the normal RVGLS group (7.15 ± 3.55 days vs. 5.79 ± 4.45 days), although this difference did not reach statistical significance (*P* = 0.145). However, prolonged hospitalizations exceeding 7 days (50.0% vs. 28.8%, *P* = 0.044) and 10 days (21.4% vs. 5.8%, *P* = 0.029) were significantly more frequent in the reduced RVGLS group. A higher proportion of patients with reduced RVGLS required oxygen at admission (78.6% vs. 55.8%, *P* = 0.043), and the duration of oxygen therapy was also longer (4.30 ± 3.27 days vs. 2.60 ± 3.82 days, *P* = 0.043). However, no significant difference was observed between groups regarding oxygen requirement at discharge (10.7% vs. 8.0%, *P* = 0.613). We conducted a multivariate analysis incorporating RVGLS and adjusting for age, hemoglobin level, heart rate, and NT-proBNP. In this model, none of the evaluated parameters, including RVGLS (B = –0.056, *P* = 0.521), demonstrated an independent association with prolonged hospitalization.

Radiological patterns did not differ significantly between groups, although lobar pneumonia was more frequent in the reduced RVGLS group (78.6% vs. 63.5%, *P* = 0.164). Intensive care unit (ICU) admission rates were similar between the two groups (7.1% vs. 5.8%, *P* = 0.560). There were no significant differences in in-hospital mortality (3.6% vs. 3.8%, *P* = 0.732), 7-day mortality (3.6% vs. 0.0%, *P* = 0.342), or 30-day mortality (3.6% vs. 3.8%, *P* = 0.732) between patients with reduced and normal RVGLS (Table 4).

**Table 4:** Clinical outcomes according to RVGLS status (*n* = 80)

Variable	Reduced RVGLS (*n* = 28)	Normal RVGLS (*n* = 52)	*P*-value
Length of hospital stay (day)	7.15 ± 3.55	5.79 ± 4.45	0.145
O_2_ therapy duration (day)	4.30 ± 3.27	2.60 ± 3.82	0.043
Radiological pattern			0.164
Lobar	22 (78.6)	33 (63.5)	
Multilobar	6 (21.4)	19 (36.5)	
O_2_ requirement			
At admission	22 (78.6)	29 (55.8)	0.043
At discharge	3 (10.7)	4 (7.7	0.613
Hospitalization			
> 7 Days	14 (50.0)	15 (28.8)	0.044
> 10 Days	6 (21.4)	3 (5.8)	0.029
ICU admission	2 (7.1)	3 (5.8)	0.560
In-hospital mortality	1 (3.6)	2 (3.8)	0.732
7-Day mortality	1 (3.6)	0	0.342
30-Day mortality	1 (3.6)	2 (3.8)	0.732

Values are presented as mean ± standard deviation or number (%). Continuous variables were compared using the independent samples t-test. Categorical variables were compared using the chi-square test or the Fisher exact test, with the Fisher exact test applied when expected cell counts were low

*ICU* Intensive care unit, *RVGLS* right ventricular global longitudinal strain

## Discussion

Determining the threshold for reduced RVGLS is an important aspect of interpretation, as no universally validated cutoff exists and normal values may vary by age, sex, and population. To define an appropriate threshold for our cohort, we reviewed previous studies evaluating RV strain in both clinical and healthy populations. In hospitalized patients with COVID-19 pneumonia, mean RVGLS values among those without adverse outcomes were − 20.3% ± 6.1%, with significant differences across outcome groups [3]. Likewise, a study of 276 healthy volunteers reported mean RVGLS values of − 24.7% ± 2.6% in men and − 26.7% ± 3.1% in women, with the lower limit of normal approximated at − 20% [4]. Across the literature, lower-limit values generally cluster near − 20% [8, 9]. Therefore, to maintain consistency with published data, we adopted − 20% as the threshold to categorize RVGLS values in our study.

One of our main findings was that reduced RVGLS was significantly associated with prolonged hospitalization exceeding seven days. Patients with reduced RVGLS also more frequently required oxygen therapy at admission and had longer durations of oxygen support during hospitalization. The association between reduced RVGLS and prolonged hospitalization observed in univariate analysis warrants careful interpretation, particularly regarding whether RVGLS reflects a marker of illness severity or a direct causal determinant of clinical outcomes. In more severe pneumonia or early septic physiology, heightened inflammatory activity may generate a hyperdynamic circulatory state that adversely influences RV longitudinal mechanics. Prior studies have likewise demonstrated associations between elevated C-reactive protein (CRP) levels, impaired RVGLS, and greater disease severity [10, 11].

In our cohort, CRP levels and radiological findings did not differ significantly between reduced and normal RVGLS groups. Taken together, these findings suggest that the RVGLS differences we observed are not fully explained by measured inflammatory or radiologic markers alone. Instead, reduced RVGLS may function as an integrative marker of early cardiopulmonary stress and overall disease burden, identifying patients at risk for increased morbidity, such as higher oxygen requirements and delayed recovery, even when conventional echocardiographic parameters remain within normal limits.

The clinical relevance of prolonged hospitalization further highlights the importance of early risk stratification. Prolonged inpatient stays impose a substantial economic burden on healthcare systems. In previous cost-analysis studies, prolonged hospitalization in CAP has been shown to impose a substantial economic burden, with average inpatient costs exceeding £3,400 in the United Kingdom and clear incremental increases with comorbid cardiac disease, while even a 1-day reduction in length of stay was associated with meaningful cost savings [12, 13]. Moreover, patients with extended hospital stays consistently exhibited more severe pneumonia and higher short-term post-discharge mortality. In this context, identifying patients at risk for prolonged hospitalization may facilitate closer monitoring and targeted interventions. Our findings suggest that RVGLS is a valuable parameter to support such predictions.

RV strain is inherently load dependent and may be influenced by preload, venous return, and inflammatory hyperdynamic states. To minimize potential confounding, we assessed intravascular volume using vena cava inferior (VCI) evaluation and excluded patients with clinical congestion. Although VCI and physical examination are not gold-standard measures of volume status, this approach reflects routine clinical practice and provides a reasonable estimate of preload in this setting. NT-proBNP is known to increase with age and varies by sex, with men exhibiting higher reference limits [14]. It is also correlated with heart rate and indices of RV systolic performance [15, 16]. Troponin levels may similarly rise in the setting of tachycardia, systemic infection, RV strain, or hypoxemia [17, 18]. Importantly, patients with known left-sided heart failure or significant diastolic dysfunction were excluded from this study, supporting the interpretation that biomarker elevations, as well as RV-related differences, even when within the conventional reference range (e.g., TAPSE, RVEF, RV and pulmonary artery diameters), more likely reflect early RV systolic impairment or subclinical pulmonary hypertension rather than overt RV failure [7, 19]. Alternatively, these biomarker changes could be attributed to myocardial stress and higher heart rates at the time of echocardiography, rather than heart failure with preserved ejection fraction. This pattern aligns with the occurrence of reduced RVGLS despite normal conventional echocardiographic parameters and identifies RVGLS as a sensitive marker of subtle RV impairment.

Despite the observed differences in clinical presentation and severity, ICU admission rates and mortality outcomes (in-hospital, 7- and 30-day mortality) did not differ significantly between groups in this study. This finding indicates that while reduced RVGLS is associated with greater morbidity, it may not necessarily translate into increased short-term mortality within this cohort. However, interpretations of adverse outcomes must consider the characteristics of our study population. The exclusion of major cardiovascular comorbidities, which are among the strongest predictors of pneumonia-related mortality, resulted in a lower-risk cohort, contributing to the small number of adverse events (three deaths overall). This selection effect, combined with limited event numbers, reduces the statistical power to detect differences in ICU utilization or mortality. Therefore, the lack of statistically significant differences should be interpreted accordingly.

### Limitations

This study has several limitations that should be acknowledged when interpreting its findings. First, although patients with clinically overt heart failure were excluded, the possibility of preexisting RV dysfunction or subclinical cardiopulmonary conditions influencing RVGLS or biomarker levels cannot be completely ruled out. Similarly, while we attempted to minimize the impact of preload by excluding patients with clinical congestion and assessing volume status using VCI measurements, these methods do not represent gold-standard assessments of intravascular volume. Therefore, residual preload- or flow-related effects on RV strain are possible, particularly in a pneumonia cohort prone to variable hemodynamic states.

Second, the sample size was small, particularly regarding adverse outcomes such as ICU admission and mortality. The exclusion of major cardiovascular comorbidities contributed to a lower-risk study population, which likely reduced the number of events. Consequently, the statistical power to detect differences in rarer outcomes is limited, and null findings, especially in multivariate models, should be interpreted with caution. Finally, follow-up was limited to in-hospital and short-term outcomes. Thus, the long-term prognostic implications of reduced RVGLS, including potential effects on post-discharge recovery, readmission risk, or long-term cardiopulmonary function, remain unknown and warrant further investigation.

## Conclusions

Overall, our findings highlight that RVGLS may serve as a valuable non-invasive marker to identify patients at risk for a more severe clinical course and prolonged hospitalization even in the absence of clinically overt RV failure. This finding aligns with prior evidence linking subclinical RV dysfunction to adverse outcomes in respiratory illness and emphasizes the importance of incorporating RV functional assessment into routine echocardiographic evaluation.

## Data Availability

The data that support the findings of this study are not publicly available due to privacy and ethical restrictions, as they contain sensitive patient information. However, the data are available from the corresponding author upon reasonable request.

## Abbreviations

BNP: Brain natriuretic peptide
CAP: Community-acquired pneumonia
CRP: C-reactive protein
ICU: Intensive care unit
IQR: Interquartile range
RV: Right ventricular
RVGLS: Right ventricular global longitudinal strain
TAPSE: Tricuspid annular plane systolic excursion
VCI: Vena cava inferior
